# Ocular Manifestations in Hematological Disorders

**DOI:** 10.7759/cureus.27941

**Published:** 2022-08-12

**Authors:** Mohamed Bouazza, Houda Youssefi, Nouama Bouanani

**Affiliations:** 1 Department of Ophthalmology, Faculty of Medicine, Mohammed VI University of Health Sciences (UM6SS), Casablanca, MAR; 2 Department of Hematology, Faculty of Medicine, Mohammed VI University of Health Sciences (UM6SS), Casablanca, MAR

**Keywords:** ocular manifestations, retinal hemorrhage, dry eye disease, multiple myeloma, non-hodgkin's lymphoma, acute myeloid leukemia

## Abstract

Background

Ophthalmic manifestations are a common feature in hematological malignancies and may be divided into two groups: those attributable to the disease's infiltration of the eye, and the ocular consequences due to blood abnormalities. This study aims to determine the prevalence of ocular manifestations and their association with the different hematological disease subgroups.

Materials and methods

We conducted a retrospective and observational study enrolling 137 consecutive patients on active treatment or follow-up for hematological malignancies from January 2016 to January 2020 at the Cheikh Khalifa International University Hospital. All patients underwent a standardized and comprehensive ophthalmic evaluation.

Results

Ocular involvement was primarily disclosed in acute myeloid leukemia (AML), non-Hodgkin's lymphoma (NHL), and multiple myeloma (MM). We herein report the different ocular changes divided into anterior segment manifestations, posterior segment manifestations, dry eye disease, and ocular adnexa findings. Patients with leukemia had a significantly higher rate of lesions in the ocular posterior segment (p < 0.001). Despite the high prevalence of anterior segment and ocular adnexa findings in lymphoma patients, no significant association emerged between these lesions and the aforementioned condition. In addition, dry eye disease was found in all instances without any association with the disease itself.

Conclusions

Awareness of ocular pathology in hematological malignancies is important as it may precede systemic diagnosis or be a sign of recurrence. We should also be concerned about the side effects of treatments, predominantly, dry eye disease. Therefore, periodic ophthalmic assessment throughout the disease's course, as well as interdisciplinary coordination of care, is crucial to promote early diagnosis and treatment, hence improving long-term outcomes.

## Introduction

Hematological diseases can lead to ocular manifestations in up to 90% of patients [1]. To date, there is scant literature regarding publications on ocular involvement and hematological diseases. This may be due to the fact that they are rare compared to other ocular diseases and hardly lead to severe complications. These abnormalities may arise as an initial manifestation of an underlying hematological disorder or be present during the disease’s course progression or treatment.

In most cases, the ocular surface and retina are the most involved structures in hematological malignancies such as leukemia, Hodgkin and non-Hodgkin lymphoma, myeloproliferative and myelodysplastic syndromes. Common findings include conjunctival hemorrhages, intraretinal hemorrhages, and cotton wool spots. The authors herein describe and assess the different ocular manifestations observed in 137 patients followed for hematological malignancies.

## Materials and methods

This is a cross-sectional retrospective observational study, jointly conducted by the ophthalmology and the hematology departments at the Cheikh Khalifa International University Hospital. Consecutive cases were collected over a period of four years between January 2016 and January 2020, including 137 patients with confirmed hematologic malignancies, divided into groups according to their underlying disease. Patients with coexisting ocular or systemic diseases, such as HIV, diabetes, and hypertension, as well as patients with ocular-orbital tumors, were ruled out based on specific predetermined criteria. Severely ill patients who were unable to consent to examination and pediatric patients under the age of 18 were also excluded. In regards to systemic diseases, exclusion criteria were positive diagnostic serological testing for HIV, fasting blood glucose of ≥ 126 mg/dl, two-hour blood glucose of ≥ 200 mg/dl, HbA1c of ≥ 6.5%, systolic blood pressure of ≥ 140 mmHg and/or diastolic blood pressure of ≥ 90 mmHg and patients under treatment for arterial hypertension. The diagnosis of ocular-orbital tumors was based on the history, examination, and neuroimaging using a computerized tomography (CT) scan. Data collection was done according to a preset format, including a demographic profile of the patient, medical history, and a record of hematological data. A complete eye examination was conducted, including a visual acuity assessment (VA) using the Snellen chart, and slit lamp biomicroscopy for the anterior and posterior segment evaluation. VA < 20/40 was considered visual loss (VL). After pupillary dilatation with 1% Tropicamide eye drops, the posterior segment was examined using indirect ophthalmoscopy. Images of the anterior and posterior segments were taken solely for documentation purposes unless the patient was unable to cooperate for clinical pictures. Intraocular pressure (IOP) was measured using an applanation tonometer. IOP equal to or greater than 21 mmHg was deemed compatible with ocular hypertension. Diagnosis and staging of dry eye disease (DED) were performed according to the Dry Eye WorkShop criteria, ocular surface disease index, tear production by the 5-min Schirmer test, break-up time, and ocular surface staining. The Schirmer test was performed using sterile strips with anesthetic, and the break-up time was measured after cornea coloration by fluorescence. In debilitated patients, the initial examination was done bedside, evaluating the anterior chamber with a portable slit-lamp biomicroscope and the posterior chamber with direct ophthalmoscopy, after pupillary dilatation. If required, patients were then evaluated in the outpatient department.

The study was approved by the Institutional Ethics Board of the Cheikh Khalifa Zaid International University Hospital. Patient consent was waived as the study included only unidentifiable data, in accordance with national law. Interpretation and analysis of the acquired results were carried out using IBM SPSS (IBM Corp., Armonk, NY) for descriptive statistics. The association between ocular manifestations and hematological parameters was checked by Chi-square analysis and Fisher test. The overall significance level was set at 95% with p < 0.05 taken as statistically significant.

## Results

The study included 137 cases of hematological malignancies with an overall median age of 55.9 years, a relevant male predominance (63.5%), and a sex ratio of 1.74. Acute myeloid leukemia, non-Hodgkin's lymphoma, and multiple myeloma were the most frequent hematological malignancies among our patients. Ocular manifestations were present in 82 patients (59.8%) and absent in 55 patients (40.1%) (Table 1).

**Table 1:** Hematological disorders distribution and the presence of ocular manifestations

	Hematological disorders	Overall (n=137)	Ocular manifestations (n=82)
Leukemia		41	31 (75.6%)
	Acute Lymphocytic Leukemia	8	6 (75%)
	Acute Myeloid Leukemia	25	19 (76%)
	Chronic Myeloid Leukemia	3	3 (100%)
	Chronic Lymphocytic Leukemia	3	2 (66%)
	Chronic Myelomonocytic Leukemia	1	1 (100%)
	Plasma Cell Leukemia	1	0 (0%)
Lymphoma		39	23 (58.9%)
	Non-Hodgkin's Lymphoma	23	15 (65.2%)
	Hodgkin's Lymphoma	13	7 (53.8%)
	T-cell Lymphoma	3	1 (33.3%)
Multiple Myeloma		26	15 (57.6%)
Other		31	13 (41.9%)
	Myelofibrosis	2	0 (0%)
	Essential Thrombocythemia	4	2 (50%)
	Waldenström Macroglobulinemia	2	1 (50%)
	Primary Polycythemia (Vaquez disease)	9	4 (44.4%)
	Myelodysplastic Syndrome	11	3 (27.3%)
	Bone Marrow Failure	3	3 (100%)

**Figure 1:**
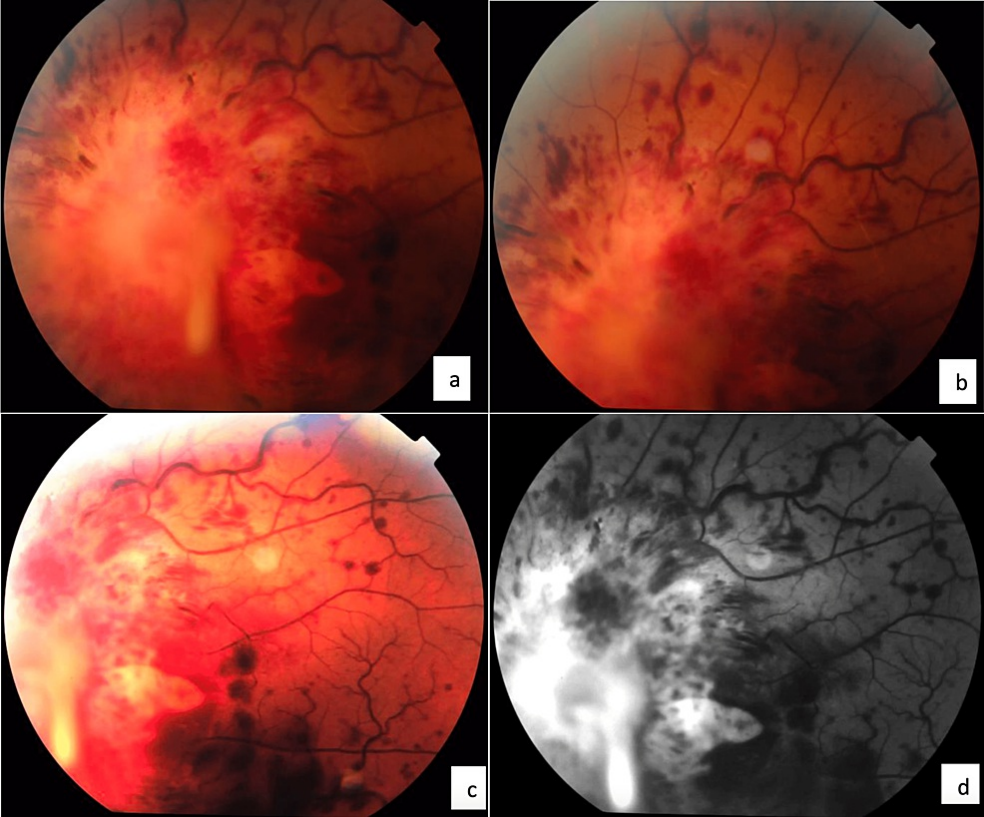
Retinograms before (a, b, c) and after fluorescein injection (d) showing retinal hemorrhages in the setting of combined central retinal vein and artery occlusion associated with massive tumor infiltration of the optic nerve in a patient followed for acute lymphoblastic leukemia.

**Figure 2:**
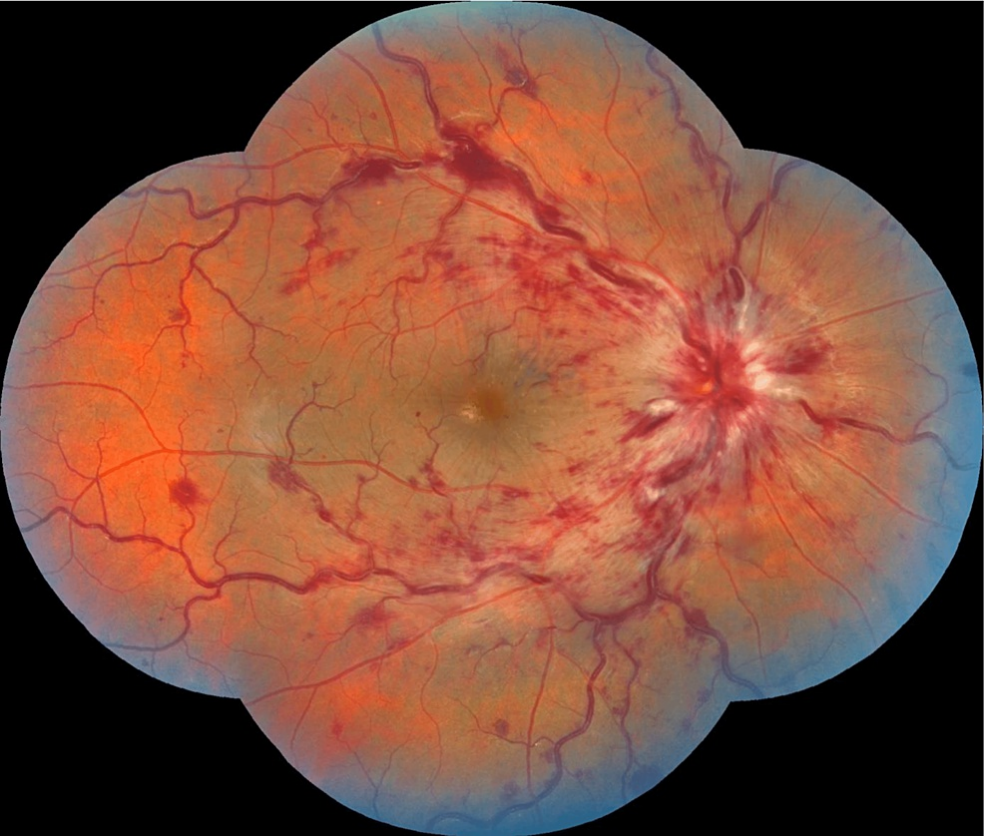
Retinography showing signs of central retinal vein occlusion: papilledema, venous tortuosity, deep and flaming retinal hemorrhages, cotton nodules in a patient followed for acute myeloid leukemia.

**Figure 3:**
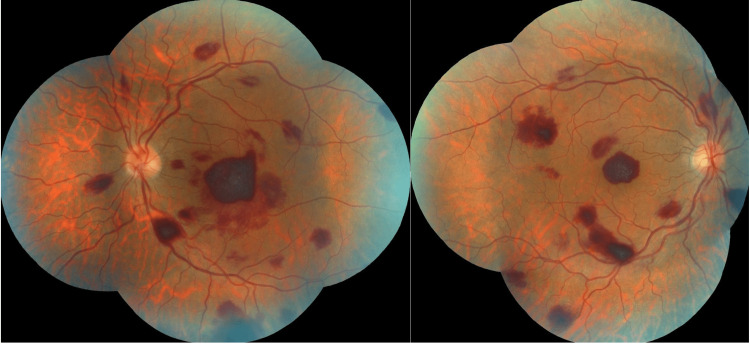
Multiple bilateral retinal hemorrhages in a patient followed for acute myeloid leukemia.

Different ocular lesions were reported, with retinal hemorrhage and dry eye disease being the most common manifestations (Figures 1-3).

**Figure 4:**
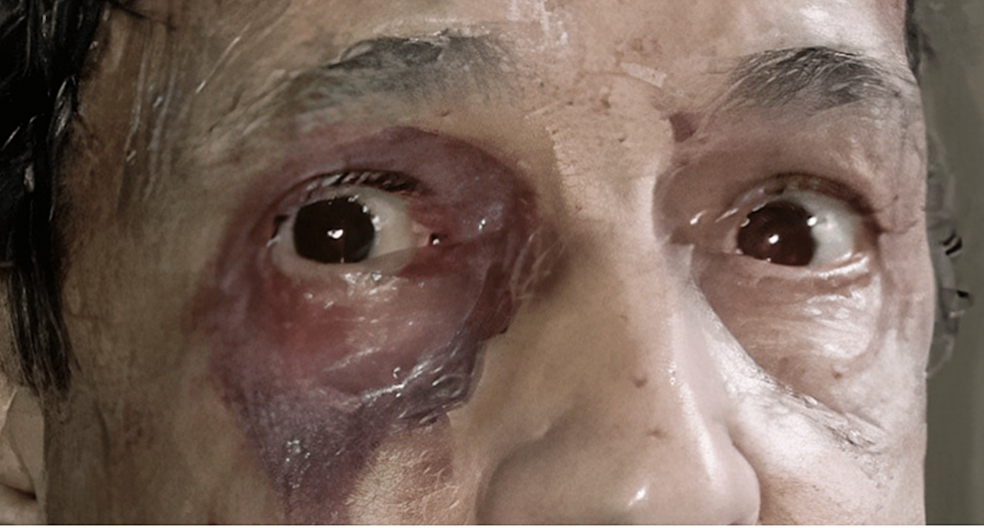
Orbital amyloidosis in a patient with multiple myeloma.

**Figure 5:**
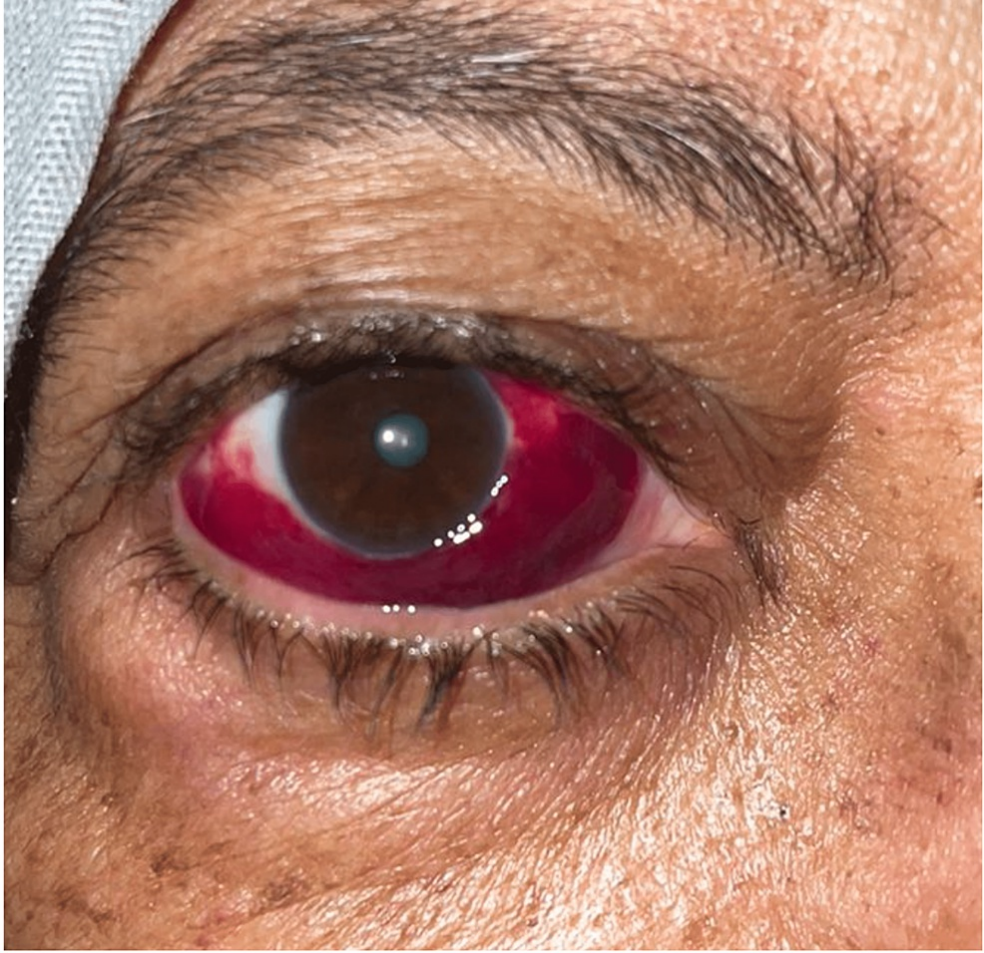
Subconjunctival hemorrhage in a patient with acute myeloblastic leukemia.

**Figure 6:**
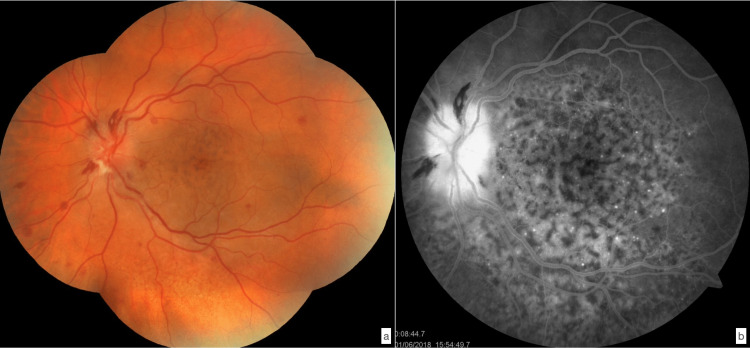
a- Retinography showing papilledema, multiple retinal hemorrhages and Roth spots in a 58-year-old patient followed for a Philadelphia-negative myeloproliferative syndrome upgraded to acute myeloblastic leukemia. b- After fluorescein injection: papilledema, pinpoint and “salt and pepper” pigmentary mottling.

Furthermore, ocular lesions were found to be highly correlated with the presence of hematological malignancies (p < 0.005). Ocular manifestations were classified into four subgroups: dry eye disease, anterior segment (Figures 4, 5), posterior segment (Figure 6), and ocular adnexa involvement (Table 2).

**Table 2:** Ocular findings in 137 patients with hematological malignancies.

	Hematological disorders	Dry eye disease (n=47)	Anterior segment (n=41)	Posterior segment (n=71)	Ocular adnexa (n=35)
Leukemia							13	8	55	12
	Acute Lymphocytic Leukemia	3	2	18	3
	Acute Myeloid Leukemia	8	6	36	4
	Chronic Myeloid Leukemia	1	0	0	3
	Chronic Lymphocytic Leukemia	1	0	0	2
	Chronic Myelomonocytic Leukemia	0	0	1	0
	Plasma Cell Leukemia	0	0	0	0
Lymphoma		16	16	0	20
	Non-Hodgkin's Lymphoma	10	11	0	14
	Hodgkin's Lymphoma	5	5	0	5
	T-cell Lymphoma	1	0	0	1
Multiple Myeloma		10	12	10	3
Other		8	5	6	0
	Myelofibrosis	0	0	0	0
	Essential Thrombocythemia	0	0	0	0
	Waldenström Macroglobulinemia	1	0	1	0
	Primary Polycythemia	4	4	2	0
	Myelodysplastic Syndrome	3	1	0	0
	Bone Marrow Failure	0	0	3	0

Dry eye disease is a non-specific and common manifestation occurring in all hematological malignancies during the treatment or follow-up phase, and no association with any particular group was found. The DEWS-modified stage was used to diagnose DED and stage it as a severity score ranging from 1 to 4. No severe cases corresponding to DEWS score 4 were found, whereas 21 patients were assigned a DEWS score of 1, 15 a DEWS score of 2, and 11 a DEWS score of 3. A high prevalence of DED was found in the non-Hodgkin's lymphoma and multiple myeloma populations. The distribution of DED patients across all diseases is tabulated in Table 2. In addition, posterior segment lesions were particularly found in leukemia patients, with a high correlation found between the two (p < 0.001). Conversely, the high incidence of anterior segment lesions in patients with lymphoma showed no correlation.

Patients were further classified according to their ocular lesions (Table 3). Posterior segment pathology such as superficial and deep retinal hemorrhages and Roth spots were the most common ophthalmic features (80%), significantly associated with acute leukemia (p < 0.005). The latter may be secondary to rheological changes in the blood rather than due to direct tumor infiltration.

**Table 3:** Types of ocular findings in 137 patients with hematological malignancies. AML = acute myeloid leukemia; ALL = acute lymphocytic leukemia; CML = chronic myeloid leukemia; CLL = chronic lymphocytic leukemia; CMML = chronic myelomonocytic leukemia; PCL = plasma cell leukemia; MM = multiple myeloma; MDS = myelodysplastic syndrome; PV = polycythemia vera (primary polycythemia); WMG = waldenström macroglobulinemia; ET = essential thrombocythemia; MF = myelofibrosis; NHL = non-Hodgkin's lymphoma; HL = Hodgkin's lymphoma; ATL = adult T-cell lymphoma; BMF = bone marrow failure; HZO = herpes zoster ophthalmicus; CRVO = central retinal vein occlusion.

Ocular manifestations		AML	ALL	CML	CLL	CMML	PCL	MM	MDS	PV	WMG	ET	MF	NHL	HL	ATL	BMF
Dry eye		8	3	1	1	-	-	10	3	4	1	-	-	10	5	1	-
Anterior segment	HZO	-	-	-	-	-	-	2	-	-	-	-	-	-	-	-	-
	Corneal ulcers	2	1	-	-	-	-	3	-	-	-	-	-	4	-	-	-
	Periorbital amyloidosis	-	-	-	-	-	-	1	-	-	-	-	-	-	-	-	-
	Subconjunctival hemorrhage	4	1	-	-	-	-	6	1	4	-	-	-	7	5	-	-
Posterior segment	Retinal hemorrhage	13	8	-	-	1	-	6	-	-	1	-	-	-	-	-	3
	Cotton wool spots	1	-	-	-	-	-	-	-	-	-	-	-	-	-	-	-
	Roth spots	14	6	-	-	-	-	-	-	-	-	-	-	-	-	-	-
	CRVO	4	1	-	-	-	-	2	-	2	-	-	-	-	-	-	-
Ocular adnexa	Ptosis	1	1	-	-	-	-	-	-	-	-	-	-	2	-	-	-
	Strabismus	-	1	-	-	-	-	1	-	-	-	-	-	4	1	-	-
	Nystagmus	-	-	-	-	-	-	-	-	-	-	-	-	-	1	-	-
	Exophthalmos	-	-	-	-	-	-	-	-	-	-	-	-	3	-	1	-
	Palpebral edema	-		3	-	-	-	-	-	-	-	-	-	1	2	-	-
	Conjunctivitis	3	1	-	2	-	-	2	-	-	-	-	-	4	1	-	-

## Discussion

In the present study, dry eye disease (DED) was the most prevalent ocular manifestation, ranging from mild to moderate cases, whereas no severe cases were found. DED is a debilitating and potentially sight-threatening condition. The recent Dry Eye Workshop II (DEWS II) report launched by the Tear Film & Ocular Surface Society (TFOS) defines DED as a multifactorial ocular surface disease characterized by a loss of the tear film homeostasis. The definition also encompasses ocular symptoms as a central feature, including discomfort, visual disturbance, or both, as well as the etiological roles of hyperosmolarity, ocular surface inflammation, damage, and neurosensory abnormalities [2]. In many studies, patients with hematological malignancies undergoing chemotherapy, total body irradiation treatments, or hematopoietic stem cell transplantation (HSCT) frequently displayed ocular surface impairment. As yet, DED is the hallmark of chronic ocular graft-versus-host disease, a major iatrogenic complication of allo-HSCT [3]. In contrast, little information is available about DED prior to the different treatment options which may underestimate its real prevalence at this stage. Therefore, a comprehensive ophthalmic evaluation of pre- and post-transplantation is required to reevaluate the real prevalence of ocular surface impairment. In fact, a study of 203 hematological patients undergoing HSCT found that DED was significantly present before transplantation. These findings support a recent recommendation that an ophthalmological evaluation should be part of the HSCT protocol [4]. In a multidisciplinary approach, early detection and treatment of DED are essential for minimizing ocular damage. The three-pronged treatment modalities include decreasing ocular surface inflammation, lubrication and tear preservation, and prevention and control of tear evaporation. Topical lubrification remains the first line of treatment, with the use of tear substitutes supplemented with viscous ointments. Various pharmacological therapies, dietary and environmental changes, as well as eyewear and contact lenses, are some of the treatment modalities offered [5].

Posterior segment manifestations were also most prominent in our study, including retinal hemorrhage, cotton wool spots, Roth spots, and central retinal vein occlusion, particularly in patients with leukemia. Visual loss, ocular history of scotomas, and eye floaters were the most common symptoms for referral to ophthalmic examination. The aforementioned signs were present either as a first manifestation of the disease, early worsening, or as a sign of relapse. Leukemic ophthalmopathy can either be “primary” through direct infiltration or “secondary” to systemic conditions, opportunistic infections, or chemotherapy-related. Primary invasion of ocular tissue by leukemic cells may occasionally result in proptosis, cranial nerve palsy, optic nerve and extensive choroidal infiltration, and exudative retinal detachment. Immunosuppression caused by illness or its treatment, such as chemotherapy and bone marrow transplantation, can lead to opportunistic infections [6,7]. The overwhelming majority of patients with leukemia reported in our survey involved retinal hemorrhages (65%), similarly described in Bukhari et al. retrospective cross-sectional study including 81 acute leukemia patients, with 60% of patients presenting ocular manifestations [8]. Male showed a greater predisposition, possibly due to the protective effect of estrogen in women against retinopathy [9]. In addition, the majority of the ophthalmic manifestations were secondary to rheological changes in blood, affecting mainly the posterior structures far more often than the anterior segment. Variables such as age, type of leukemia, staging, and response to systemic chemotherapy or bone marrow transplantation might influence ocular symptoms. In addition, leukemic ophthalmopathy was found to be more common in acute and myeloid cases compared to chronic and lymphoid subtypes. Ocular lesions are typically proportional to the disease's severity, although they can sometimes be the presenting sign of leukemia or may signify an isolated focal relapse after complete recovery from systemic leukemia. Similarly, amongst our patients was one case of refractory acute lymphocytic leukemia (ALL) revealed by strabismus. Involvement of the central nervous system (CNS), namely cranial nerve palsy, is more prevalent in ALL patients than in AML patients [10, 11]. In addition, a high proportion of patients with acute leukemia harbor ophthalmic manifestations, a majority of which can be prevented by maintaining Hb levels >7 g/L and platelet counts >50,000 cells/mm^3^ [12]. Hence, these patients should undergo an ocular examination at baseline and frequently thereafter to check for the development of new symptoms. On the basis of their interpretations of ocular symptoms and blood counts, ophthalmologists should prescribe therapy adjustments such as blood transfusions in collaboration with hematologists.

Conversely, patients with multiple myeloma presented lesions occurring in nearly every ocular structure. Infiltrations of the orbit, conjunctiva, uvea, lacrimal sac, and lacrimal gland have been documented. Additional ocular signs included chorioretinopathy, neuro-ophthalmic abnormalities, corneal deposits, and opportunistic infections. Notably, orbital involvement occurs more commonly than intra-ocular involvement [13]. A recent study evaluated the potential association between ocular disorders and specific multiple myeloma therapies by conducting a full ophthalmic assessment on 93 multiple myeloma patients undergoing active treatment or follow-up after various treatment lines. Lens opacities and dry eye syndrome were the most detected ocular features. The study concluded that there was no reliable correlation between multiple myeloma treatments and ocular disorders [14]. In a previous study based on the Swedish medical registries, Hemminki et al. observed that senile cataract and glaucoma were the most prevalent visual manifestations in patients with multiple myeloma, with a sharply rising frequency beyond age 60. The authors attributed this discovery to the deposition of M-protein and light chains in the eye lens, a tissue with a greater protein concentration than other tissues. This environment may promote protein aggregation, resulting in opacification of the ocular lens and restriction of humoral outflow in the iridocorneal angle [15].

In our study, patients with Hodgkin’s and non-Hodgkin’s lymphomas had exclusively ocular anterior segment lesions, whereas no ocular posterior segment findings were noted in these subgroups. The most common presentation was subconjunctival hemorrhage without a characteristic pattern. Amongst our patients, was a rare case of a 52-year-old patient with nodular sclerosing Hodgkin’s lymphoma, revealed by a peripheral vestibular syndrome and nystagmus. As yet, there is scant literature regarding the association of both nystagmus and Hodgkin’s lymphoma [16]. Ophthalmic manifestations in systemic lymphoma may be the result of direct infiltration invading the optic nerve or the vitreoretinal, corneal or conjunctival inflammation, paraneoplastic retinopathy, or drug-induced lesions [17]. Moreover, locations of ophthalmic lymphomas might be intraocular or adnexal. Ocular appendages, consisting of visual accessory structures, may seldom be implicated in malignant hematopoietic proliferation. Although any form of lymphoma can affect the orbit, the vast majority of ocular adnexal lymphomas (OALs) are non-Hodgkin's lymphomas (NHL). In ophthalmologic practice, Hodgkin's lymphoma is exceedingly rare. The clinical symptoms and signs of OAL are non-specific and overlap with those of several other orbital illnesses, which can result in delaying the diagnosis. Although OAL patients have a low mortality rate, untreated cases can result in blindness [18].

Despite including accurate institutional databases of a relatively large sample, the study is primarily limited by its retrospective approach. In addition, the exclusion of patients due to frailty may hinder our ability to fully report the ocular manifestations in our series. These observations require more investigation within the framework of a prospective study.

## Conclusions

This study reports and assesses ocular pathological findings in patients followed for hematological malignancies. Various cases highlight the ability of these hematological diseases to manifest ocular involvement as well as important clinical findings that may be observed with each. This was notably exemplified by the presence of posterior segment lesions in patients with leukemia.

Furthermore, with the advent of novel antineoplastic therapies that may prolong life expectancy, these instances demonstrate the significance of eye care for patients with hematologic malignancies, particularly dry eye disease. Thus, our findings support periodic ophthalmic assessment before and throughout therapy, as well as the follow-up, in order to optimize supportive care and improve the quality of life.

## References

[1] Lang GE, Lang SJ (2011). Ocular findings in hematological diseases [Article in German]. Ophthalmologe.

[2] Craig JP, Nichols KK, Akpek EK (2017). TFOS DEWS II definition and classification report. Ocul Surf.

[3] Giannaccare G, Bonifazi F, Sessa M (2017). Ocular surface analysis in hematological patients before and after allogeneic hematopoietic stem cell transplantation: implication for daily clinical practice. Eye (Lond).

[4] Giannaccare G, Bonifazi F, Sessa M, Fresina M, Arpinati M, Bandini G, Versura P (2016). Dry eye disease is already present in hematological patients before hematopoietic stem cell transplantation. Cornea.

[5] Nair S, Vanathi M, Mukhija R, Tandon R, Jain S, Ogawa Y (2021). Update on ocular graft-versus-host disease. Indian J Ophthalmol.

[6] Orhan B, Malbora B, Akça Bayar S, Avcı Z, Alioğlu B, Özbek N (2016). Ophthalmologic findings in children with leukemia: a single-center study. Turk J Ophthalmol.

[7] Skarsgård LS, Andersson MK, Persson M, Larsen AC, Coupland SE, Stenman G, Heegaard S (2019). Clinical and genomic features of adult and paediatric acute leukaemias with ophthalmic manifestations. BMJ Open Ophthalmol.

[8] Bukhari ZM, Alzahrani A, Alqarni MS, Alajmi RS, Alzahrani A, Almarzouki H, Alqahtani AS (2021). Ophthalmic manifestations in acute leukemia patients and their relation with hematological parameters in a tertiary care center. Cureus.

[9] Nuzzi R, Scalabrin S, Becco A, Panzica G (2018). Gonadal hormones and retinal disorders: a review. Front Endocrinol (Lausanne).

[10] Fozza C, Dore F, Isoni MA (2014). Strabismus and diplopia in a patient with acute myeloid leukemia. Am J Case Rep.

[11] Vishnevskia-Dai V, Sella King S, Lekach R, Fabian ID, Zloto O (2020). Ocular manifestations of leukemia and results of treatment with intravitreal methotrexate. Sci Rep.

[12] Soman S, Kasturi N, Srinivasan R, Vinod KV (2018). Ocular manifestations in leukemias and their correlation with hematologic parameters at a tertiary care setting in South India. Ophthalmol Retina.

[13] Chin KJ, Kempin S, Milman T, Finger PT (2011). Ocular manifestations of multiple myeloma: three cases and a review of the literature. Optometry.

[14] Pennisi M, Berchicci L, Miserocchi E (2019). Ocular disorders in multiple myeloma patients: cross-sectional study of prevalence and association with treatment. Leuk Lymphoma.

[15] Hemminki K, Försti A, Tuuminen R (2016). The incidence of senile cataract and glaucoma is increased in patients with plasma cell dyscrasias: etiologic implications. Sci Rep.

[16] Bouanani N, Aasfara J, Hajjij A, Ouhabi H (2020). Hodgkin's lymphoma presenting with an unusual horizontal Nystagmus and vertigo. Tunis Med.

[17] Valenzuela J, Echegaray JJ, Dodds E, Kurup SK, Lowder C, Ondrejka SL, Singh AD (2021). Ophthalmic manifestations of Hodgkin lymphoma: a review. Ocul Oncol Pathol.

[18] Mulay K, Honavar SG (2016). An update on ocular adnexal lymphoma. Semin Diagn Pathol.

